# International perspective on healthcare provider gender bias in musculoskeletal pain management: a scoping review

**DOI:** 10.1136/bmjopen-2025-107766

**Published:** 2026-01-12

**Authors:** Katherine F Wilford, Maria Jesus Mena-Iturriaga, Margaret Vugrin, Macarena Wainer, Gesine H Seeber

**Affiliations:** 1 Physical Therapy Program, Murphy Deming College of Health Sciences, Mary Baldwin University, Fishersville, Virginia, USA; 2 Facultad de Medicina Clínica Alemana, Universidad del Desarrollo, Santiago, Santiago Metropolitan Region, Chile; 3 Preston Smith Library, Texas Tech University Health Sciences Center, Lubbock, Texas, USA; 4 School of Medicine and Health Sciences, Division of Orthopedics at Campus Pius-Hospital, Carl von Ossietzky Universität Oldenburg, Oldenburg, Germany; 5 University Medical Center Groningen, Department of Orthopedics, University of Groningen, Groningen, The Netherlands

**Keywords:** PAIN MANAGEMENT, Musculoskeletal disorders, Quality in health care

## Abstract

**Abstract:**

**Objective:**

Musculoskeletal pain is a global issue affecting millions of individuals. Healthcare provider gender bias (HCP-GB) in pain management or treatment may have implications. This study aimed to systematically (1) identify and map the scientific and grey literature as it relates to HCP-GB in the assessment, diagnosis and management of musculoskeletal pain, and (2) identify current gaps that necessitate further research.

**Design:**

This scoping review was conducted in accordance with the Preferred Reporting Items for Systematic Reviews and Meta-Analyses extension for Scoping Reviews (PRISMA-ScR).

**Data sources:**

The following databases were searched: PubMed (National Library of Medicine), Embase (Elsevier), Scopus (Elsevier), CINAHL Complete (Ovid), Academic Search Complete (EBSCOhost), Pre-Prints Database (National Library of Medicine) and Rehabilitation Reference Center from inception to August 2022 and updated in May 2025. Relevant grey literature was identified.

**Eligibility criteria for selecting articles:**

All screening was performed by two reviewers during title/abstract screening and full-text screening stages. Articles published in English, Spanish and German were included if they involved participants with musculoskeletal pain and examined HCP-GB as the dependent variable.

**Data extraction and synthesis:**

Two reviewers independently extracted data from the bibliometric, study characteristics and pain science variables. Results were descriptively mapped, and the frequency of concepts, population and characteristics was narratively reported.

**Results:**

21 full-text articles were included. All articles were published in North America and Europe. A total of 3694 healthcare providers from various specialty areas were examined. A majority of studies (57.1%; n=12) measured HCP-GB using written case vignettes, 33.3% (n=7) used case vignettes plus virtual human pictures/videos, and 9.5% (n=2) used real patients. The influence of patients’ sex in HCP pain assessment was reported in 28.5% (n=6) of the articles, while 42.9% (n=9) reported gender bias regarding HCP non-pharmacological treatment recommendations. Male patients were more likely to receive exercise recommendations for back pain and laboratory testing, whereas female patients received more psychological treatment recommendations and counselling from their HCP.

**Conclusions:**

While there appears to be inconsistent use of the terms sex and gender, the literature informing this review suggests an existence of gender bias in the management of patients with musculoskeletal pain. Future research should be more purposeful in the use of sex/gender-related terms and consider exploring the impact of implicit bias training to rectify potential gender biases present in HCP.

**STRENGTHS AND LIMITATIONS OF THIS STUDY**

- The research team comprises an international group of researchers as well as a scientific librarian with expertise in developing search strategies.
- The protocol for this scoping review has been previously peer reviewed and published.
- A web-based collaboration software platform (Covidence) was used during the screening and data extraction processes for optimal adherence to best practice guidelines in the conducting and reporting of scoping reviews.
- While this scoping review focused on gender bias, it is difficult to separate the intersectionality of gender with other biological or societal constructs.
- Only references in English, Spanish and German were included, which may have inadvertently excluded a wider variety of studies that could have improved the diversity of findings.

## Introduction

 According to the *International Association of the Study of Pain,* pain is ‘an unpleasant sensory and emotional experience associated with, or resembling that associated with, actual or potential tissue damage.’^1^ Worldwide, pain conditions such as low back and neck pain and other musculoskeletal (MSK) disorders are reported among the primary reasons for lost working years due to disability.^2 3^ With up to US$635 billion of costs annually, including US$300 billion only for direct healthcare costs, pain poses a considerable economic burden on society.^4^ According to current projections, until the year 2050, the prevalence of MSK pain will increase substantially, placing additional strain on already overburdened healthcare systems.^3^

Notably, pain goes beyond its economic impact, as it also has widespread societal implications. Chronic pain can have substantial socio-economic consequences by limiting affected individuals’ ability to work.^5^ Moreover, pain can impact affected individuals’ relationships, decrease their self-esteem and increase their susceptibility to mental health disorders such as anxiety, catastrophising and depression.^5^ Approximately 20% of individuals experiencing pain that persists for longer than 3 months (ie, chronic pain) express suicidal thoughts, where the incidence rate ratio of suicide attempt is four times higher than in the general population.^6^,^8^ In the primary healthcare setting, patients with chronic pain are frequently prescribed opioids, estimating that approximately 10% of these patients develop an opioid use disorder including substance abuse and/or overdose.^59^,^15^ Thus, practitioners who treat patients with MSK pain are obliged to continuously assess and adapt their management approach to adequately address their patients’ pain and prevent its far-reaching repercussions on individuals and in society.

Effectively managing individuals with pain requires a thorough consideration of the healthcare-patient management model, which includes multiple facets, such as examination, evaluation, diagnosis, prognosis, intervention and outcomes.^16^ This process begins with obtaining a comprehensive patient history to gather relevant data about their current condition, followed by the use of various tests and measures to identify impairments more objectively.^17^ Healthcare providers (HCP) must carefully analyse the collected data to formulate an accurate diagnosis and prognosis based on the information obtained during that examination phase. Following this, a comprehensive management strategy for the patient should be devised.^17^ It is crucial to avoid any data collection or interpretation inaccuracies during the process, as they may lead to suboptimal or adverse clinical decisions, compromising patient outcomes.

When evaluating individuals with MSK pain, the primary diagnostic benchmark relies on patients’ self-reported symptoms^18 19^ where information can be obtained through verbal communication during medical history-taking or via written patient self-reported outcome measures.^16^ However, it is essential to recognise that communication between the clinician and patient during the history-taking process can be vulnerable to various sources of error, such as bias.^20^ Bias refers to a predisposition or inclination towards an object or person, which can manifest in positive or negative forms.^21^ Biases frequently originate from stereotypes rather than factual understanding and can potentially drive imprudent decisions or foster discriminatory practices.^21^ Biases against others often hinge on the group to which individuals belong or on inherent physical attributes such as ethnicity, sexual orientation, age or gender.^18 21 22^

It is possible for individuals to exhibit bias without being aware of their predispositions. Such unconscious bias, also referred to as implicit bias, can have negative adverse effects as stereotypes and prejudgement can significantly influence an individual’s views and decisions. Despite comprehensive training in specialised domains, HCP may exhibit implicit biases. The interaction between patients and HCP is a complex process in which scientific evidence, clinical expertise and patient expectations play a role, but may not always be reconciled. It is imperative that HCPs are aware of the potential for implicit bias and reflect on the impact this may have on their patients when translating clinical expertise and research into patient care.

While there are several areas where an implicit bias may occur, gender bias is one that has received attention recently.^23 24^ Literature indicates gender-based disparities in the evaluation, diagnosis and treatment of pain.^18 22 25 26^ Women/females frequently encounter delays in accessing diagnostic and therapeutic interventions for pain compared with men/males. Additionally, they are less likely to receive prescriptions for pain-relieving medications or referrals for radiological assessments and physical therapy.^27^,^29^ Currently, there is a lack of a comprehensive and systematic literature review on implicit gender bias in healthcare. Although an informed review on gender bias in pain treatment was published in 2018,^26^ there is a need for a more expansive exploration that explicitly addresses implicit gender bias in healthcare. Additionally, previous literature has not covered the entire patient management model nor focused solely on MSK pain. Therefore, this scoping review’s purpose is to identify and describe the available scientific and grey literature on healthcare provider gender bias (HCP-GB) in the evaluation, diagnosis and treatment of MSK pain. Specifically, this study aims to (1) examine the available literature on HCP-GB in MSK pain assessment, diagnosis and management, (2) analyse the impact of HCP-GB on patient outcomes and (3) investigate the variations of HCP-GB across different countries and cultures. Throughout this scoping review, the term gender will refer to a social concept associated with psychological, cultural and/or behavioural roles/identities that originate within the society in which an individual resides.^30^,^32^ Whereas an individual’s sex often pertains to the physical and physiological characteristics, including hormones, gene expression and reproductive anatomy.^30^,^32^ This distinction aims to clarify the healthcare implications of gender by emphasising cultural ramifications rather than solely biological distinctions.

## Methods

### Study design

This scoping review was conducted in accordance with current recommendations from the Joanna Briggs Institute and the Preferred Reporting Items for Systematic Reviews and Meta-Analyses extension for Scoping Reviews (PRISMA-ScR).^33^,^36^ Accordingly, this scoping review’s protocol was published prior to the final search and data extraction.^16^

### Data sources and search strategies

Applicable scientific literature was identified via the following electronic databases from inception to 23 August 2022: PubMed (National Library of Medicine), Embase (Elsevier), Scopus (Elsevier), CINAHL Complete (Ovid), Academic Search Complete (EBSCOhost), Pre-Prints Database (National Library of Medicine) and Rehabilitation Reference Center. Grey literature was identified by systematically searching Trip Database, Papers First, Conference Papers Index and Clinical Trial Register Databases (PROSPERO, ClinicalTrials.gov, WHO International Clinical Trials Registry Platform (ICTRP), ISRCTN registry, ClinicalResearch.com, CenterWatch) and Google Scholar. Due to the high volume of references from the first search iteration and the time required to screen, the search was updated on 13 May 2025. The full search strategies for major databases can be found in online supplemental appendix A.

With the assistance of an experienced scientific librarian (MV), the final search strategy was developed using the P.C.C. framework illustrated in table 1.^33 35^ This strategy used a combination of medical subject headings and concepts subject headings, modified based on the individual databases. Additionally, the snowball method was used to identify other relevant sources from the included studies’ reference lists. Due to the skills of the author team, resources were deemed eligible if they were published in English, Spanish or German.^37^,^39^ References from scientific literature and grey literature were included if they involved human participants with MSK pain and examined HCP-GB as the dependent variable. All methodologies were included; however, reviews/meta-analyses were excluded from data extraction to avoid extracting duplicate data.

**Table 1:** P.C.C. framework

**P**opulation	Man, woman, adult, healthcare provider, professional
**C**oncept	Bias, gender, stereotype, gendered norm, inequity, sex, gender research
**C**ontext	Musculoskeletal pain, pain assessment, pain perception, treatment, pain management, rehabilitation, diagnosis, outcome, culture, equity in health

### Article selection

The review management software Covidence (Veritas Health Innovation, Melbourne, Australia) was used for article screening, selection and data extraction. Before starting the final review, two pilot screenings were incorporated, where the first aimed to define and revise the search criteria and the second at refining article selection and data extraction processes.^33 35 40^ A three-reviewer model was employed throughout the entire article selection process, where two reviewers (KFW and MW) independently and blinded to the other reviewer’s vote, screened each item. In case of conflicts between both reviewers’ votes, a third reviewer (GHS) resolved these.

During the title/abstract and full-text screening phases, all types of research methodology were included. During the data extraction phase, however, any review (ie, umbrella, systematic, narrative) and meta-analyses were excluded. Subsequently, their reference lists were screened for additional suitable references by one reviewer (GHS). Relevant resources identified were uploaded into Covidence and screened using the previously described process.

### Study variables

Bibliometric and study characteristics data, as well as pain science variables, were extracted from the selected full-text articles (table 2).

**Table 2:** Data extraction for bibliometric, study characteristics and pain science variables

	Bibliometric	Study characteristics	Pain science
Variables to be extracted and mapped	Author(s) Type of study Publication year Journal DOI Language Country	Study design Setting Sample/population Purpose(s) Aim(s) Level of evidence Approach (qualitative vs quantitative vs mixed methods)	Healthcare provider specifications: profession, age, sex, gender, degree, years of experience, country, religion, ethnic background Patient specifications: age, sex, gender, education level, socioeconomic status, country, religion, ethnic background, mental health status, comorbidities, severity of health issue, familial history, accessibility to healthcare Healthcare setting Pain descriptors: location(s), intensity, onset, type Diagnosis: outcome measures/assessment tools used, time from pain onset, timeline, sex-related prevalence, diagnostic criteria available Type of treatment prescribed: medication vs rehab vs psychological vs other Patient outcome after treatment Bias explanation(s)
Reporting measures	Author(s), type of study, publication year, country, methodology, etc → absolute and relative frequencies Study purposes and aims → descriptively Variables related to population age and sample size → mean (SD)/median (IQR) Population gender and other characteristics → absolute or relative frequencies Pain variables → descriptively and/or as absolute or relative frequencies		

Wilford *et al*. International perspective on healthcare provider gender bias in musculoskeletal pain management: a scoping review protocol. *BMJ Open*. 2022;12(6):e059233. doi:10.1136/bmjopen-2021-059233 (used with permission).

DOI, digital object identifier; IQR, Interquartile range; SD, Standard deviation.

### Data extraction and analysis

Data were extracted into a custom-built and piloted template in the Covidence software. Extracted data were downloaded from the review manager and cross-referenced to assure accuracy. Subsequently, data were mapped and charted descriptively. Population, concept and context frequencies, as well as other details, were reported.

## Results

This scoping review’s purpose was to identify and describe the available scientific and grey literature on HCP-GB in the evaluation, diagnosis and treatment of MSK pain. Specifically, this study aimed to (1) examine the available literature on HCP-GB in MSK pain assessment, diagnosis and management, (2) analyse the impact of HCP-GB on patient outcomes and (3) investigate the variations of HCP-GB across different countries and cultures.

### Article inclusion

**Figure 1:**
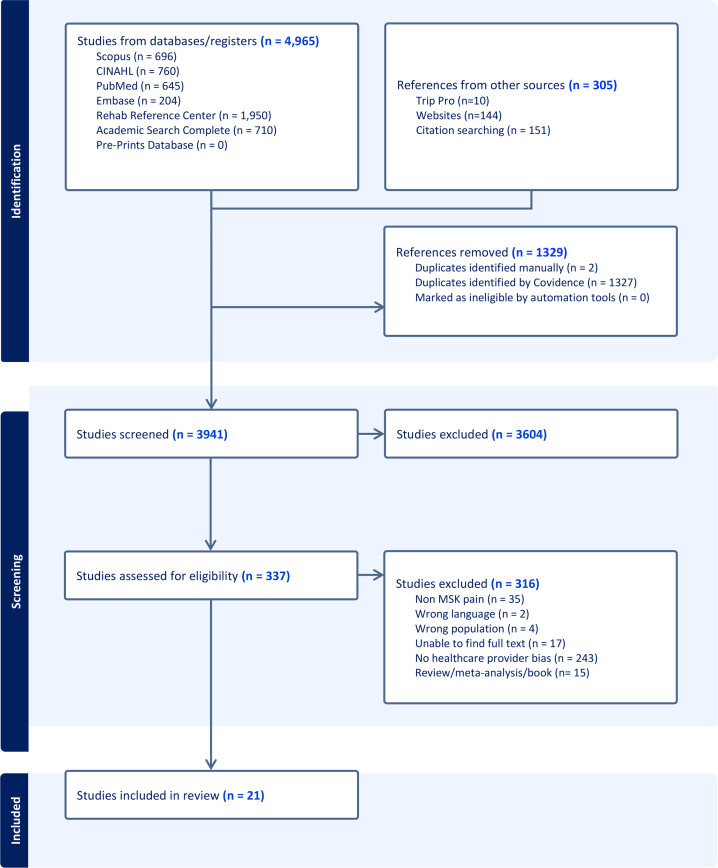
PRISMA-ScR flow chart. MSK, musculoskeletal; PRISMA-ScR, Preferred Reporting Items for Systematic Reviews and Meta-Analyses extension for Scoping Reviews.

The initial search resulted in 5270 references. Following manual and computer-generated deduplication (via Covidence), the title and abstract were screened for 3941 references. Of these references, 337 qualified for full-text review. Refer to figure 1 for exclusion reasons during full-text review (n=316). Data from 21 references were included to inform this scoping review.

### Characteristics of included studies

All included articles were in the English language. Cross-sectional studies and prospective cohort studies were reported in four articles (19.0%)^41^,^44^ and one (4.7%) article,^45^ respectively. Six articles (28.5%)^2746^,^50^ did not report a study design. Most articles (n=19; 90.4%) were published since 2000.^2741^,^44 46^ Fifteen (71.4%) of the articles mentioned the continent where the respective studies were performed: 10 (47.6%)^44^,^4649 50 52 54 55 58 59^ in North America and five (23.8%)^2741^,^43 53^ in Europe. The study settings most frequently reported were ‘outpatient surgery center’ and ‘emergency room’, with four (19.0%)^42 48 51 59^ and four (19.0%)^43 45 56 57^ studies, respectively. Further details regarding study characteristics are shown in table 3 and online supplemental file 1.

**Table 3:** General characteristics of the included articles (n=21)

	Frequency (%)
Language	
German	0 (0)
English	21 (100)
Spanish	0 (0)
Publication year	
2019–2025	3 (14.2)
2010–2018	11 (52.4)
2000–2009	5 (23.8)
1990–1999	1 (4.8)
<1990	1 (4.8)
Study design	
Cross-sectional	4 (19.0)
Experimental design (2×2×2 between subjects)	1 (4.7)
Experimental design (between subjects)	1 (4.7)
Prospective cohort study	1 (4.7)
Difference in pain management/strategies	8 (38.0)
Not reported	6 (28.5)
Healthcare setting	
Emergency room/department	4
Post-anaesthesia care unit	1
Outpatient surgery centre	4
Rehabilitation centre	1
Primary healthcare centre*	1
Pain specialty rehabilitation clinic*	1
Not reported	11

Data presented in absolute frequency (%).

* Multiple settings in the study.

### Healthcare providers and patient characteristics

#### Healthcare providers

A total of 3694 HCP were recruited in the 21 included studies. Of the included studies, one (4.7%)^57^ recruited only female HCP, with the other 20 articles (95.2%)^2741^,^56 58^ included male and female participants. Several studies included more than one HCP, with 17 (80.9%),^2741^,^45 47^ six (28.5%),^27 41 45 55 58 59^ nine (42.9%)^4243 45^,^47 55 56 58 59^ and three (14.3%)^42 57 59^ of the studies including physicians, medical students, nursing professionals and nursing students, respectively. Dentists were included in two (9.5%)^49 50^ studies whereas nutritionists,^52^ physical and occupational therapists^53^ and psychologists^53^ were included in one (4.7%) study each. In terms of reported experience, four (19.0%)^42^,^4451^ of the studies included HCP with <10 years of professional experience, five (23.8%)^47 49 51 52 57^ studies included HCP with 10–20 years of professional experience and one (4.7%)^27^ article reported professional experience greater than 20 years. Eight (38.0%)^4546 53^,^55 58^ articles reported HCP’s ethnic background, with a majority of included HCP who identified as white. Further details regarding the HCP characteristics are shown in online supplemental file 1.

Regarding the contexts in which bias was measured, 12 (57.1%)^41^,^4346 48 50^ studies used only written case vignettes to describe the patients and provide clinical context. Seven (33.3%)^27 47 49 50 55 58 59^ studies used case vignettes plus virtual human pictures or videos, which allowed the manipulation of different patient features to create high-fidelity variations in pain expression and demographic characteristics. The videos included in one of those studies showed faces of patients with shoulder pain during painful physiotherapeutic manoeuvres.^27^ Two studies (9.5%)^45 53^ used real patients.

#### Patient characteristics, pain description, diagnosis and treatment

In four (19%)^49 50 55 58^ articles, the sex/gender vocabulary was correctly used, referring to ‘gender; male; female; no binary’. Weiner *et al* was the only author that described a patient’s socioeconomic status as a characteristic.^51^ Information about the pain onset was described in most of the included articles (n=15; 71.4%),^41^,^4345^ and details about patients’ pain intensity were reported in five (23.8%)^43^,^4652^ articles. Patients’ medical diagnosis was reported in four (19.0%)^44^,^4657^ of the articles. The patient’s pain location was detailed in 20 (95.2%)^2741^,^52 54^ of the articles, with the lower back being the most frequent pain region (n=16; 76.2%).^42^,^4547^ Further details regarding the patient characteristics are shown in online supplemental file 2.

### Outcomes of included articles

Six (28.5%)^27 41 43 45 56 60^ articles reported on the influence of patients’ sex on HCP pain assessment. HCPs tend to judge pain differently in men and women, with women’s pain more likely to be perceived as exaggerated, emotionally triggered, psychosomatic in nature, unspecific, less credible, less urgent and severe^27 41 47 56^—especially in the case of acute pain and with lacking evidence of pathology.^27 60^ Moreover, HCP expressed the need for diagnostic support from other HCP more often with female patients.^41^ In contrast, one study found that HCP perceived females as credible in their increased pain experience.^45^

Nine (42.9%)^27 41 44 45 48 51 52 54 55^ articles informing this review reported on gender bias regarding HCP non-pharmacological treatment recommendations. According to these, HCP tend to recommend different non-pharmacological treatments for men and women, with male patients being more likely to receive general and specific exercise recommendations for selected back pain disorders,^48 51^ as well as more laboratory testing.^41^ In contrast, female patients receive more psychological treatment recommendations^27 55^ and, in the case of low back pain, obtain more counselling from their HCP, including home chores and pelvic floor exercises,^44^ versus male patients.

For the HCP characteristics and their approach to patients, 10 (47.6%)^41 43 45 46 51 52 54 55 58 59^ articles reported developing a difference in pain management or strategies between males and females. Seven (33%)^27 45 46 48 52 55 58^ articles reported sex/gender bias regarding pharmacological treatment. One (4.7%)^52^ article found male patients are more likely to receive more pain medication and higher dosages when prescribed by a male HCP, whereas two (9.5%)^48 52^ articles reported female patients with persistent low back pain receive medication at higher dosages when prescribed by a female HCP. Only one (4.7%)^55^ study found that women are more likely to receive antidepressant medication compared with men. In contrast, other authors did not find sex/gender a predictive factor for the number and strength of medications prescribed.^45 46^

One (4.7%)^42^ of the articles investigated the legitimation of pain by health professionals. This study suggests that HCPs’ sexist attitudes and gender role ideology impact their willingness to support female patients presenting with low back pain.^42^

### Impact of bias

Eleven articles (52.4%)^2747^,^52 54 56 58 59^ reported exploring other constructs in addition to gender bias. Race (n=8; 38%)^47^,^5052 54 58 59^ and age (n=4; 19%)^47 49 50 54^ were the most frequently explored constructs. Other constructs included mental health status, pain characteristics, non-verbal communication and physician characteristics (table 4).

**Table 4:** Intersectional constructs

Article	Title	Other construct explored
Bartley 2015	The influence of health care professional characteristics on pain management decisions.	Race Age Physician characteristics (experience)
Bernardes 2011a	On the contextual nature of sex-related biases in pain judgments: The effects of pain duration, patient’s distress and judge’s sex	Pain (duration)
Boissoneault 2016	Assessment of the Influence of Demographic and Professional Characteristics on Health Care Providers’ Pain Management Decisions Using Virtual Humans.	Race Age
Green 2003	Clinical decision making in pain management: Contributions of physician and patient characteristics to variations in practice.	Race Age Pain (type) Physician characteristics
Hirsh 2013	The influence of patient’s sex, race and depression on clinician pain treatment decisions.	Race Mental health (depression)
Hollingshead 2015	Impact of race and sex on pain management by medical trainees: a mixed methods pilot study of decision making and awareness of influence.	Race
Schäfer 2016	Health care providers’ judgments in chronic pain: the influence of gender and trustworthiness.	Mental health (depression)
Wandner 2014	The impact of patients’ gender, race, and age on health care professionals’ pain management decisions: an online survey using virtual human technology.	Race Age
Weiner 2011	Managing nonspecific low back pain: do nonclinical patient characteristics matter?	Socioeconomic status
Weisse 2001	Do gender and race affect decisions about pain management?	Race
Weisse 2003	The influence of gender and race on physicians’ pain management decisions.	Race

## Discussion

Despite the high prevalence of MSK pain, there is simultaneously a lack of a comprehensive and systematic literature review on HCP-GB in this population. Specifically, this study aimed to (1) examine the available literature on HCP-GB in MSK pain assessment, diagnosis and management, (2) analyse the impact of HCP-GB on patient outcomes and (3) investigate the variations of HCP-GB across different countries and cultures.

A notable aspect that emerged from this scoping review pertained to the utilisation of gendered language in research studies examining sex/gender-related bias in the management of patients with MSK pain. It was observed that most authors frequently employed the terms man/male and woman/female interchangeably.^2741^,^48 51^ This practice, however, is problematic as it conflates the concepts of sex and gender. Despite the inherent interconnectedness of the two constructs, they remain distinct entities.^61^ As previously delineated, sex refers to the biological and physiological characteristics that define humans as male, female or intersex, whereas gender encompasses the social, cultural and psychological aspects of being a man, woman or non-binary individual.^62^ It is imperative to differentiate between sex and gender in relevant scientific literature for several reasons. First, it enables healthcare professionals to identify and comprehend the discrete effects of biological sex and gender identity on health outcomes, including MSK pain management. For instance, biological sex can influence the expression and severity of certain conditions, such as hormonal differences affecting pain perception.^63^ In contrast, gender identity can impact how individuals experience and report pain, as well as their interactions with HCP.^64^ The employment of precise terminology by HCP enables the formulation of care plans that consider these differences and the development of targeted interventions to address specific patient needs. Second, the failure to differentiate between the terms sex and gender can perpetuate existing biases and stereotypes, potentially leading to inaccurate and misleading conclusions. For instance, the assumption that all individuals who identify as women will experience symptoms such as MSK pain in the same way can overlook the diversity of experiences within this group and neglect the needs of individuals who do not conform to traditional gender norms. Third, precise terminology can facilitate effective communication between HCP and patients, ensuring that both parties have a shared understanding of the concepts being discussed. By recognising and respecting the complexity of gender identity and enhancing clarity in communication, HCP can develop more inclusive and effective management strategies tailored to the unique needs of diverse patient populations.

The current scoping review included studies that examined bias as the dependent variable. By doing so, the impact of intersectionality on the included studies’ outcomes was minimised. Additionally, this allowed data to be extracted from a variety of study designs and resources in the current project. A previous systematic review about racial bias evaluated implicit bias in created scenarios.^65^ This systematic review reported that HCPs’ unconscious attitudes and associations regarding race/ethnicity may influence their behaviour without conscious awareness.^65^ Furthermore, those authors concluded that future research should address concerns surrounding implicit bias, such as in a real clinical context that may increase HCPs’ stress and negatively influence implicit bias.^65^ While there is literature to support the use of implicit bias training courses for HCP, a widespread, structured and standardised approach to this type of training is currently lacking.^66 67^ Future research in this area is warranted to ensure optimal outcomes for both the HCP and the patients managed by these professionals. Examining implicit bias among HCP, as opposed to only looking at differences in clinical management, would offer a more comprehensive approach to understanding the impact of biases.

Regarding the influence of bias on patient management and outcomes, the results of this scoping review support the notion of a gender bias on behalf of the included HCP.^27 41 45 56 60^ The symptoms of women were more likely to be dismissed as having a physical or biological origin and more likely to be categorised as psychological in nature.^27 41 47 56 60^ The potential consequences of these findings could influence the management of patients with MSK pain. For example, previous research has shown that women are less likely to be prescribed medication for pain-related symptoms when compared with men.^68^

The geographical distributions of articles in this scoping review reveal a significant lack of diversity. The paucity of representation from regions such as Asia, Africa, South America and the Middle East decreases the generalisability of the findings. This limits the understanding of the cultural influence in the healthcare setting. It is, thus, challenging to determine whether the observed gender biases are universal or culturally specific to North America and Europe since expectations around pain expression, gender roles and trust in medical professionals may vary across societies. To comprehensively understand the global scope of HCP-GB, future research must include and consider diverse socio-cultural and geographical settings, thus allowing a broader and inclusive understanding of gender bias in MSK pain care.

While this scoping review has several strengths, including a diverse team of researchers from three continents and an experienced scientific librarian, it is not without limitations. First, only references in English, Spanish or German were included. This may have inadvertently excluded a wider variety of studies that could have improved the diversity of findings. Second, true to the nature of scoping reviews, the methodological quality of the included articles was not assessed. Therefore, the extracted data should be interpreted with caution. Finally, the current scoping review only included references wherein gender bias was examined as the dependent variable. This resulted in many studies using written patient scenarios versus real-world patients. While a clinical scenario may not mimic the true clinical behaviour of HCP, it enables researchers to gain a clearer understanding of HCP’s unconscious attitudes and associations regarding gender. Future studies should consider comparing the results of implicit bias testing to real-world clinical outcomes.

## Conclusion

This scoping review systematically mapped the available evidence on HCP-GB in the management of patients with MSK pain. This study explored the influence of bias on the diagnosis and treatment of MSK pain in a variety of healthcare settings and from a variety of HCP. While there appears to be inconsistent use of the terms sex and gender, the literature informing this review suggests an existence of gender bias in the management of patients with MSK pain. Future research should be more purposeful in the use of sex/gender-related terms and consider exploring the impact of implicit bias training to identify and rectify potential gender biases present in HCP.

## Supplementary material

10.1136/bmjopen-2025-107766 online supplemental file 1

10.1136/bmjopen-2025-107766 online supplemental file 2

10.1136/bmjopen-2025-107766 online supplemental file 3

## Data Availability

All data generated or analysed during this study are included in this published article.
