# Supplementary material for: International perspective on healthcare provider gender bias in musculoskeletal pain management: a scoping review
Source: BMJ Open. 2026 Jan 12;16(1):e107766. doi: 10.1136/bmjopen-2025-107766 (PMC12815234; doi:10.1136/bmjopen-2025-107766)
Supplement: online supplemental file 1 [file bmjopen-16-1-s001.docx]

**Supplementary File 1.** Study specifications and Health care provider characteristics.

| **Study Specifications** | | | | **Health Care Provider Characteristics** | | | | | |
| --- | --- | --- | --- | --- | --- | --- | --- | --- | --- |
| **Author, Year** | **Sample Size** | **Context** | **Country** | **Sex/**  **Gender** | **Age** | **Profession** | **Reported experience** | **Religion** | **Ethnic background** |
| AlSad  2022 | N=58 HCP | Case Vignettes | United States | Men and female | < 45 years (n=39) | Generalist medical practitioners; Practitioners from Women's health, sports medicine, osteopathic manipulation | 33 HCP (56.9%)  < 10 YoPE | NR | - 78% White  - 10% Asian  - 7% Black or African -American  - 2% Middle Eastern/North African  - 3% Other |
| Bartley  2015 | N=154 HCP | Case Vignettes and Virtual Human | United States | Men and female | 46.6 years (SD=12.9) | Dentists; Physicians | 16.8 YoPE  (SD=13.7) on average | NR | - 68.8% (106) Caucasian  - 48 No Caucasian:  - 39.6% Asian  - 20.8% Black/ African American  -22.9% Hispanic  -16.7% Other |
| Bernardes 2011a | N=205 HCP | Case Vignettes | NR | Men and women | Mean 20.64 years (SD=2.7) | Nursing students | At the time of the study:  - 48% of students were finishing their 1^st^ year of formal training;  - 26.1% were finishing their 2^nd^ year:  - remaining students were finishing their 3^rd^ or 4^th^ years. | NR | NR |
| Bernardes 2011b | N=126 | Case Vignettes | NR | Female | Mean 35.33 years, SD=7.64 (22-60) | Nursing professionals | Mean 11.82 (SD=7.12); (1-34 YoPE) | NR | NR |
| Bernstein 1981 | N=253 | Case Vignettes | NR | Man and woman | Mean M: 45 years;  Mean F: 41 years | Generalist medical practitioners | NR | NR | NR |
| Boissoneault 2016 | N=152 HCP | Case Vignettes and Virtual Human | United States | Man and woman | 46.52 years  (SD=12.97) | Dentists; Physicians | 16.99 YoPE  (SD=13.60) | NR | - 69% White,  - 11.8% Asian  - 7.2% Hispanic  - 6.6% Black/African American  - 5.3% other |
| Criste  2003 | N=133 HCP | Case Vignettes | United  States | Men and female | NR | Certified registered nurse anesthetists | NR | NR | NR |
| Green  2003 | N=368 HCP | Case Vignettes | United States | Men, female | Mean 45 years (SD=13) | Generalist as well as Specialist medical practitioners | NR | NR | - 80% White;  - 4.1% Black;  - 2.2% Hispanic/Latino;  - 11.6% Asian/Pacific Islander;  - 1.9% Other |
| Hamberg 2002 | N=239 | Case Vignettes | Sweden | Men and women | Mean 33 years, (range 26-56) | Medical Students/AT-physicians | Novice (interns) | NR | NR |
| Hirsh  2013 | N=100 | Case Vignettes and Virtual Human | United States | Men and women | Mean 28.28 years, (SD=6.17) | Generalist medical practitioners; Nursing professionals; Medical students, Graduate nursing students | NR | NR | Non-Hispanic  - 71% White, 71  - 20% Asian, 20  - 3% Black, 3  - 1% American Indian/  Alaska Native 1  - 5% other. 5 |
| Hirsh  2014 | N=98 HCP | Case Vignettes and Virtual Human | United States | Men and female | Mean 28.22 years (SD=6.20) | Generalist as well as Specialist medical practitioners; Nursing professionals; Medical students | NR | NR | - 97% Non Hispanic:  - 70% White  - 20% Asian  - 3% Black,  - 1% American Indian/Alaska Native  - 5% other |
| Hollingshead 2015 | N=20 HCP | Case Vignettes and Virtual Human | United States | Men and female | NR | Nursing professionals; Medical trainees (medical students, residents, fellows) | NR | NR | - 95% non-Hispanic  - 70% White  - 20% Asian  - 5% Black  - 5% Middle Eastern |
| Lehti  2017 | N=30 HCP; N=10 patients | Real patient(s) | Sweden | Men and women | Range 27-63 | Physician, Physical Therapist, Social worker, Occupational Therapist, and Psychologist | NR | NR | NR |
| Prego-Jimenez 2022 | N=80 HCP | Case Vignettes | Spain | Men and female | Mean 32.16 years (SD=10.06) | Generalist medical practitioners; Nursing professionals; Nursing students | Mean 7.60 YoPE  (SD 7.83) | NR | NR |
| Raftery 1995 | N=84 HCP | Real patient(s) | United States | Men and female | Mean M: 29.4 years (SD=3.7),  Mean F: 30.5 years (SD=5.5) | Practitioners from emergency medicine, internal medicine, and surgery, Medical student, Nurse practitioner | NR | NR | NR |
| Schäfer 2016 | N=63 HCP (N=34 pain clinicians and N=29 medical students) | Case Vignettes and videos showing human pained face | United Kingdom | Men and female | NR | Specialist medical practitioners; Medical students | Pain clinicians: most had >20 YoPE;  Medical students: most were 4th year students | NR | NR |
| Schilter  2024 | N=231 HCP | Case Vignettes | Switzerland | Men and female | Mean 35.6 years (SD=7.9) | Physicians | 5 YoPE | NR | NR |
| Wandner 2014 | N=193 HCP (N=80 physicians, N=113 nurses) | Case Vignettes / Virtual Human and Virtual Human Video | NR | Man and woman | Mean 44 years (range 22 – 75) | Generalist medical practitioners; Nursing professionals | 15 YoPE  (range: 1-47) on average | NR | - 69% Caucasian  - 11% African American,  - 6% Hispanic,  - 10% Asian,  - 4% other. |
| Weiner 2011 | N=284 | Case Vignettes | NR | Men and women | NR | Practitioners from internal medicine and emergency medicine | 217 HCP=0-5 YoPE;  17 HCP=5-10 YoPE;  28 HCP > 10 YoPE | NR | NR |
| Weisse 2001 | N=111 HCP | Case Vignettes | United States | Men and female | Mean 41.31 years (26-72) | Dietician and nutritionists; Primary care physicians (family practitioners and general internists) | 12.2 YoPE (98% of physicians) | NR | - 79% (88) White  - 2.7% (3) Black  - 2.7% (3) Hispanic  - 13.5% (15) Asian/ Pacific Islander |
| Weisse  2003 | N=712 HCP | Case Vignettes | NR | Men and female | Mean 42.4 years | Practitioners from Internal medicine, family medicine, and medical pediatric physicians | 12.2 YoPE on average | NR | - 83.4% White;  - 3.5% Black;  - 2.2% Hispanic;  - 6.2% Asian Pacific Islander  - 1.7% Other |

YoPE= years of professional experience; SD=Standard deviation; HCP=Health Care Provider; NR=Not Reported
