# Supplementary material for: International perspective on healthcare provider gender bias in musculoskeletal pain management: a scoping review
Source: BMJ Open. 2026 Jan 12;16(1):e107766. doi: 10.1136/bmjopen-2025-107766 (PMC12815234; doi:10.1136/bmjopen-2025-107766)
Supplement: online supplemental file 2 [file bmjopen-16-1-s002.docx]

**Supplementary File 2.** Patient characteristics, pain description, diagnosis, and treatment reported in each study (n = 21)

| **Author,**  **Year** | **Patient Characteristics** | | | | | | **Pain Characteristics** | | **Results** |
| --- | --- | --- | --- | --- | --- | --- | --- | --- | --- |
|  | **Country** | **Sex/**  **Gender** | **Sample Age** | **Socio-Economic Status** | **Ethnic background** | **Mental health status** | **Pain Location** | **Pain Onset** |  |
| AlSad 2022 | NR | Male; Female | NR | NR | NR | NR | Low back | NR | - HCP disagreed that LBP differs by gender - HCP were more inclined to advise female patients on pelvic floor exercises, but their evaluation of pelvic symptoms in female LBP patients was inadequate - HCP tended to recommend home chores more for females |
| Bartley 2015 | NR | Male; Female | Adults | NR | White and Black | NR | - Lower Back (for physicians)  - Mandibular (lower) left posterior teeth (for dentists) | LBP = past year; Mandibular pain = 2 months ago | - Younger and middle-aged practitioners rated females as experiencing greater pain unpleasantness - This difference was nonsignificant among older practitioners |
| Bernardes 2011a | NR | Man; Woman | 37 years | NR | NR | Agitated and anxious vs. calm and quiet | Lower Back radiating to right lower limb | 3 days; 3 years | - Biases were observed only in acute pain or when expressed without distress - Perceptions of women's pain were viewed as less credible and their clinical situations as less severe than men's - Judgments of women's pain were uniquely influenced by these contextual variables |
| Bernardes 2011b | NR | Man; Woman | 37 years | NR | NR | NR | Chronic low-back pain | 3 years ago | - Women's pain assessments were influenced by evidence of pathology - Nurses exhibited bias against men, but only when pathology was present - The impact of distress cues less consistent |
| Bernstein 1981 | NR | Male; Female | NR | NR | NR | NR | Low back | NR | - 25% of HCP believed women overburdened their time, compared to 14% for men - Women's complaints more frequently deemed emotionally influenced and psychosomatic |
| Boissoneault 2016 | NR | Man; Woman | Younger and older adult | NR | White and Black | NR | - Lower back  - Mandibular left posterior teeth | Low back: for the past year of greater than one-year duration orofacial: started approximately two months ago | - Differences in pain intensity ratings were seen only among non-white providers - White providers had higher weights for pain unpleasantness among men - Non-white males were the exception regarding visual age cues in non-opioid analgesic decisions |
| Criste 2003 | NR | Male; Female | 35 years | NR | NR | Depression | Right knee | 30 minutes post-op | - Pain management strategies were indistinguishable between male and female anesthetists, as well as between male and female patients |
| Green 2003 | NR | Male; Female | Range=24 – 74 years | NR | NR | NR | Cesarean section, myomectomy,prostatectomy, degenerative joint disease, | Acute x3 cases, Chronic x 4 cases, cancer pain x 2 cases | - Optimal treatment more often given to men with acute postoperative or cancer pain. |
| Hamberg 2002 | Sweden | Male; Female | 34 years | NR | NR | NR | Left shoulder, right shoulder, and neck | Couple of months ago | - Non-specific somatic diagnoses, psychosocial inquiries, drug prescriptions, and referrals to physiotherapists and orthopedists were more common for female patients - Laboratory tests more frequently requested for males - Both male and female physicians contributed to gender differences - Male physicians emphasized patient compliance primarily in female patients, while female physicians did so more for males. |
| Hirsh  2013 | NR | Man; Woman | NR | NR | White and Black | With or without presence of depressive symptoms (2 groups) | Low back | 1 year ago | - Patients' depression status had most significant and consistent impact on treatment decisions - While less influential overall, patients' sex and race were notably significant for a subset of participants |
| Hirsh 2014 | NR | Male; Female | NR | NR | NR | Depression^§^ | Chronic LBP | NR | - Female patients received significantly higher antidepressant and mental health referral ratings - This difference was observed only among female providers - Controlling for provider sexism scores did not change these findings, suggesting female providers are more likely to recommend psychosocial treatments for female pain patients, regardless of their sexist attitudes |
| Hollingshead 2015 | NR | Male; Female | NR | NR | White and Black | NR | Lower Back | 1 year | - Patient sex accounted for less than 1% to 27% of the variance in opioid ratings and up to 21% in antidepressant ratings - No significant sex differences were observed in average physical therapy ratings |
| Lehti  2017 | Sweden | Man; Woman | range 35-65 | NR | NR | NR | NR | NR | - Gender and sociocultural context may contribute to advantages and disadvantages during patient journeys from primary health care to a pain rehabilitation clinic - Patients and professionals perceived pain as a low-ranking illness - Women and men used different gendered strategies to legitimize pain and be taken seriously - Being ‘a proper patient ready to change’ and having ‘likeness’ between patients and professionals were viewed as advantageous - Patients with higher educational levels were perceived as easier to interact with and had better access to health care. - Professional thoughts gender norms influenced the rehabilitation options - The rehabilitation program was seen by several professionals to be better suited for women than men, which could lead to unequal care. |
| Prego-Jimenez 2022 | NR | Man/Male; Woman/  Female | NR | NR | NR | NR | Low back | *Chronic" | - The legitimation of LBP negatively correlated with gender role ideology and sexism scales when the virtual patient was female - Sexism and gender role ideology may reduce the willingness to offer support and credibility specifically for female patients. |
| Raftery 1995 | NR | Male; Female | M: 38.4 years (SD=15.0);  F: 41.9 years  (SD=16.4) | NR | White, Black, Hispanic, Asian-American, other | NR | Back, Neck, Head | Chronic (35.4% for male, 30.9% for female) or acute (64.5% for male, 69% for female) | - Female patients expressed more pain and were perceived to experience greater pain - Female patients received more medications, were less likely to receive no medication, and were prescribed more potent analgesics - Patient-reported pain was strongest predictor of medication quantity and potency, while patient gender was not a significant predictor |
| Schäfer 2016 | NR | Male; Female | 51 years (range 34- 67) | NR | NR | Without depression history; depression with onset before CP and depression onset after CP (3 groups) | Shoulder | NR | - Trustworthiness did not significantly influence pain estimates but interacted with gender - Women, particularly those viewed as low in trustworthiness, were estimated to experience less pain and seen as more likely to exaggerate it - Men were more often recommended analgesics - Women were more frequently advised psychological treatment |
| Schilter 2024 | Switzerland | Man/Male; Woman/  Female | 47 years | NR | NR | NR | Low Back Pain | 24 hour previous | - Physicians of both sexes tended to consider typical men more sensitive to pain, with less pain endurance, and more willing to report pain vs typical women. However, pain management did not differ between men and women - More referrals for women   for imaging examinations than men  Lower doses of ibuprofen and opioids prescribed for women   - The physician's gender had a modest influence on management decisions, where female physicians being more likely to prescribe ancillary examinations |
| Wandner 2014 | NR | Male; Female | Younger and older adult | NR | Caucasian or African American | NR | Lower Back | Greater than one year | - Virtual human patients who were male and African American were thought to be experiencing greater pain intensity |
| Weiner 2011 | NR | Man; Woman | 45 years | High SES (Occupation as a CEO)/ Medium SES (school teacher)/Low SES (housekeeper) | NR | NR | Low back | 2 weeks ago | - Men prescribed general conditioning exercises more often |
| Weisse 2001 | NR | Male; Female | NR | NR | White and Black | NR | Lower Back, Left buttock, and left posterior thigh | Yesterday | - No overall differences in treatment decisions or maximum permitted doses were found based on patient gender or race - Male physicians prescribed higher doses of hydrocodone to male patients, while female physicians prescribed higher doses to female patients |
| Weisse  2003 | NR | Male; Female | NR | NR | Black and White | NR | Lower Back | NR | - No overall differences in treatment decisions or maximum doses were found by gender or race - Female HCP prescribed lower doses of hydrocodone for persistent back pain, especially to male patients |

^§^status balanced across patient sex; not included in the primary statistical tests;

NR=Not Reported; HCP=Healthcare Provider; LBP=Low Back Pain; SD=Standard Deviation; M=Male; F=Female; SES=Socioeconomic Status
