# Supplementary material for: International perspective on healthcare provider gender bias in musculoskeletal pain management: a scoping review
Source: BMJ Open. 2026 Jan 12;16(1):e107766. doi: 10.1136/bmjopen-2025-107766 (PMC12815234; doi:10.1136/bmjopen-2025-107766)
Supplement: online supplemental file 3 [file bmjopen-16-1-s003.docx]

**Appendix A – Search Strategies for Major Databases**

**PubMed Search Strategy**

#3 Chronic Pain [Mesh] OR Musculoskeletal pain [Mesh] OR "Chronic Pain" [TIAB] OR "Musculoskeletal pain*" [TIAB] OR "Craniomandibular Disorders"[Mesh] OR "Temporomandibular Joint Disorders"[Mesh] OR "Temporomandibular Joint Dysfunction Syndrome"[Mesh] OR "Medial Tibial Stress Syndrome"[Mesh] OR "Myofascial Pain Syndromes"[Mesh] OR "Temporomandibular Joint Dysfunction Syndrome"[Mesh] OR "Tendinopathy"[Mesh] OR "Elbow Tendinopathy"[Mesh] OR "Tennis Elbow"[Mesh] OR "Enthesopathy"[Mesh] OR "Tendon Entrapment"[Mesh] OR "De Quervain Disease"[Mesh] OR "Trigger Finger Disorder"[Mesh] OR "Tenosynovitis"[Mesh] OR "Fibromyalgia"[Mesh] OR pain [TIAB] OR "Acute Pain"[Mesh] OR "Arthralgia"[Mesh] OR "Shoulder Pain"[Mesh] OR "Back Pain"[Mesh] OR "Failed Back Surgery Syndrome"[Mesh] OR "Low Back Pain"[Mesh] OR "Breakthrough Pain"[Mesh] OR "Facial Pain"[Mesh:NoExp] OR "Headache"[Mesh:NoExp] OR "Metatarsalgia"[Mesh] OR "Morton Neuroma"[Mesh] OR "Myalgia"[Mesh] OR "Pelvic Girdle Pain"[Mesh] OR "Neck Pain"[Mesh] OR "Piriformis Muscle Syndrome"[Mesh] OR "Pudendal Neuralgia"[Mesh] OR "Sciatica"[Mesh] OR "Nociceptive Pain"[Mesh:NoExp] OR "Pain, Intractable"[Mesh] OR "Pain, Postoperative"[Mesh] OR "Phantom Limb"[Mesh] OR "Pain, Procedural"[Mesh] OR "Pain, Referred"[Mesh] OR "Pelvic Pain"[Mesh:NoExp] OR tendinopat* [TIAB] OR Enthesopathy [TIAB] OR "tendon entrapment*" [TIAB] OR "De Quervain" [TIAB] OR "trigger finger" [TIAB] OR Tenosynovitis [TIAB] OR Fibromyalgia[TIAB] OR "acute pain" [TIAB] OR Arthralgia [TIAB] OR "Shoulder Pain"[TIAB] OR "Back Pain" OR "Failed Back Surgery" OR "Low Back Pain" OR "Breakthrough Pain" OR "Facial Pain" OR "Headache" OR "Metatarsalgia" OR "Morton Neuroma*" OR "Myalgia" OR "Pelvic Girdle Pain" OR "Neck Pain" OR "Piriformis Muscle Syndrome" OR "Pudendal Neuralgia" OR "Sciatica" OR "Nociceptive Pain" OR "Intractable pain" OR "Postoperative Pain" OR "Phantom Limb" OR "Procedural Pain" OR "Phantom Limb"[Mesh] OR "Pain, Procedural"[Mesh] OR "Pain, Referred"[Mesh] OR Pelvic Pain[TIAB]

((Delivery of Health Care [Mesh] OR Attitude of Health Personnel [Mesh] OR Emotional Intelligence [Mesh] OR Health Personnel [Mesh] OR Clinical Reasoning [Mesh] OR Judgement [Mesh] OR Trust [Mesh] OR Professional-Patient Relations [Mesh] OR "Caregivers"[Mesh] OR "Clinical Decision-Making"[Mesh] OR healthcare [TIAB] OR attitude [TIAB] OR empathy [TIAB] OR self-motivation [TIAB] OR “self-awareness” [TIAB] OR “self-regulation” [TIAB] OR “social skill*”[TIAB] OR nurse* [tiab] OR “nursing staff” [TIAB] OR Doctor* [TIAB] OR “physical therapist*” [TIAB] OR physician OR “occupational therapist” [TIAB] OR Judgement [TIAB] OR trust [TIAB] OR “Professional Patient Relationship” [TIAB] OR bias [TIAB])) AND ((Sex Factors [Mesh] OR Sexism [Mesh] OR Gender Equity [Mesh] OR Prejudice [Mesh] OR Gender Equity [Mesh] OR Gender Identity [Mesh] OR "Sex Characteristics"[Mesh] OR "Sexuality"[TIAB] OR "Gender Identity"[TIAB] OR "Sex"[TIAB] OR sexism [TIAB] OR gender [TIAB] OR "Healthcare Disparities"[Mesh])) AND (Chronic Pain [Mesh] OR Musculoskeletal pain [Mesh] OR “Chronic Pain” [TIAB] OR “Musculoskeletal pain*” [TIAB] OR "Craniomandibular Disorders"[Mesh] OR "Temporomandibular Joint Disorders"[Mesh] OR "Temporomandibular Joint Dysfunction Syndrome"[Mesh] OR "Medial Tibial Stress Syndrome"[Mesh] OR "Myofascial Pain Syndromes"[Mesh] OR "Temporomandibular Joint Dysfunction Syndrome"[Mesh] OR "Tendinopathy"[Mesh] OR "Elbow Tendinopathy"[Mesh] OR "Tennis Elbow"[Mesh] OR "Enthesopathy"[Mesh] OR "Tendon Entrapment"[Mesh] OR "De Quervain Disease"[Mesh] OR "Trigger Finger Disorder"[Mesh] OR "Tenosynovitis"[Mesh] OR "Fibromyalgia"[Mesh] OR pain [TIAB] OR "Acute Pain"[Mesh] OR "Arthralgia"[Mesh] OR "Shoulder Pain"[Mesh] OR "Back Pain"[Mesh] OR "Failed Back Surgery Syndrome"[Mesh] OR "Low Back Pain"[Mesh] OR "Breakthrough Pain"[Mesh] OR "Facial Pain"[Mesh:NoExp] OR "Headache"[Mesh:NoExp] OR "Metatarsalgia"[Mesh] OR "Morton Neuroma"[Mesh] OR "Myalgia"[Mesh] OR "Pelvic Girdle Pain"[Mesh] OR "Neck Pain"[Mesh] OR "Piriformis Muscle Syndrome"[Mesh] OR "Pudendal Neuralgia"[Mesh] OR "Sciatica"[Mesh] OR "Nociceptive Pain"[Mesh:NoExp] OR "Pain, Intractable"[Mesh] OR "Pain, Postoperative"[Mesh] OR "Phantom Limb"[Mesh] OR "Pain, Procedural"[Mesh] OR "Pain, Referred"[Mesh] OR "Pelvic Pain"[Mesh:NoExp] OR tendinopat* [TIAB] OR Enthesopathy [TIAB] OR “tendon entrapment*” [TIAB] OR “De Quervain” [TIAB] OR “trigger finger” [TIAB] OR Tenosynovitis [TIAB] OR Fibromyalgia[TIAB] OR “acute pain” [TIAB] OR Arthralgia [TIAB] OR "Shoulder Pain"[TIAB] OR "Back Pain" OR "Failed Back Surgery” OR “Low Back Pain" OR “Breakthrough Pain" OR "Facial Pain" OR "Headache" OR "Metatarsalgia" OR "Morton Neuroma*" OR "Myalgia" OR “Pelvic Girdle Pain" OR "Neck Pain" OR "Piriformis Muscle Syndrome" OR "Pudendal Neuralgia" OR "Sciatica" OR "Nociceptive Pain" OR "Intractable pain" OR "Postoperative Pain" OR "Phantom Limb" OR "Procedural Pain" OR "Phantom Limb"[Mesh] OR "Pain, Procedural"[Mesh] OR "Pain, Referred"[Mesh] OR Pelvic Pain[TIAB]) AND ("Pain Management"[Mesh])

**Embase**

('pain management'/exp OR 'pain management') AND ('delivery of health care'/exp OR 'delivery of health care' OR 'attitude of health personnel'/exp OR 'attitude of health personnel' OR 'emotional intelligence'/exp OR 'emotional intelligence' OR 'health personnel'/exp OR 'health personnel' OR 'clinical reasoning'/exp OR 'clinical reasoning' OR 'judgement'/exp OR judgement OR 'trust'/exp OR trust OR 'professional-patient relations'/exp OR 'professional-patient relations' OR 'caregivers'/exp OR caregivers OR 'clinical decision-making'/exp OR 'clinical decision-making' OR healthcare:ti,ab OR attitude:ti,ab OR empathy:ti,ab OR 'self motivation':ti,ab OR 'self awareness':ti,ab OR 'self regulation':ti,ab OR 'social skill*':ti,ab OR nurse*:ti,ab OR 'nursing staff':ti,ab OR doctor*:ti,ab OR 'physical therapist*':ti,ab OR 'physician'/exp OR physician OR 'occupational therapist':ti,ab OR judgement:ti,ab OR trust:ti,ab OR 'professional patient relationship':ti,ab OR bias:ti,ab) AND ('sex factors'/exp OR 'sex factors' OR 'sexism'/exp OR sexism OR 'prejudice'/exp OR prejudice OR 'gender equity'/exp OR 'gender equity' OR 'gender identity'/exp OR 'gender identity' OR 'sex characteristics'/exp OR 'sex characteristics' OR sexuality:ti,ab OR 'gender identity':ti,ab OR sex:ti,ab OR sexism:ti,ab OR gender:ti,ab OR 'healthcare disparities'/exp OR 'healthcare disparities') AND ('chronic pain'/exp OR 'chronic pain' OR 'musculoskeletal pain'/exp OR 'musculoskeletal pain' OR 'chronic pain':ti,ab OR 'musculoskeletal pain*':ti,ab OR 'craniomandibular disorders'/exp OR 'craniomandibular disorders' OR 'temporomandibular joint disorders'/exp OR 'temporomandibular joint disorders' OR 'medial tibial stress syndrome'/exp OR 'medial tibial stress syndrome' OR 'myofascial pain syndromes'/exp OR 'myofascial pain syndromes' OR 'temporomandibular joint dysfunction syndrome'/exp OR 'temporomandibular joint dysfunction syndrome' OR 'tendinopathy'/exp OR tendinopathy OR 'elbow tendinopathy'/exp OR 'elbow tendinopathy' OR 'tennis elbow'/exp OR 'tennis elbow' OR 'enthesopathy'/exp OR enthesopathy OR 'tendon entrapment'/exp OR 'tendon entrapment' OR 'de quervain disease'/exp OR 'de quervain disease' OR 'trigger finger disorder'/exp OR 'trigger finger disorder' OR 'tenosynovitis'/exp OR tenosynovitis OR 'fibromyalgia'/exp OR fibromyalgia OR pain:ti,ab OR 'acute pain'/exp OR 'acute pain' OR 'arthralgia'/exp OR arthralgia OR 'shoulder pain'/exp OR 'shoulder pain' OR 'failed back surgery syndrome'/exp OR 'failed back surgery syndrome' OR 'morton neuroma'/exp OR 'morton neuroma' OR 'pain, intractable'/exp OR 'pain, intractable' OR 'pain, postoperative'/exp OR 'pain, postoperative' OR 'pelvic pain'/exp OR 'pelvic pain' OR tendinopat*:ti,ab OR enthesopathy:ti,ab OR 'tendon entrapment*':ti,ab OR 'de quervain':ti,ab OR 'trigger finger':ti,ab OR tenosynovitis:ti,ab OR fibromyalgia:ti,ab OR 'acute pain':ti,ab OR arthralgia:ti,ab OR 'shoulder pain':ti,ab OR 'back pain'/exp OR 'back pain' OR 'failed back surgery'/exp OR 'failed back surgery' OR 'low back pain'/exp OR 'low back pain' OR 'breakthrough pain'/exp OR 'breakthrough pain' OR 'facial pain'/exp OR 'facial pain' OR 'headache'/exp OR headache OR 'metatarsalgia'/exp OR metatarsalgia OR 'morton neuroma*' OR 'myalgia'/exp OR myalgia OR 'pelvic girdle pain'/exp OR 'pelvic girdle pain' OR 'neck pain'/exp OR 'neck pain' OR 'piriformis muscle syndrome'/exp OR 'piriformis muscle syndrome' OR 'pudendal neuralgia'/exp OR 'pudendal neuralgia' OR 'sciatica'/exp OR sciatica OR 'nociceptive pain'/exp OR 'nociceptive pain' OR 'intractable pain'/exp OR 'intractable pain' OR 'postoperative pain'/exp OR 'postoperative pain' OR 'procedural pain'/exp OR 'procedural pain' OR 'phantom limb'/exp OR 'phantom limb' OR 'pain, procedural'/exp OR 'pain, procedural' OR 'pain, referred'/exp OR 'pain, referred' OR 'pelvic pain':ti,ab)

**SCOPUS**

(("Shoulder Pain" OR "Back Pain" OR "Failed Back Surgery Syndrome" OR "Low Back Pain" OR "Breakthrough Pain" OR "Facial Pain" OR "Headache" OR "Metatarsalgia" OR "Mortons Neuroma" OR "Myalgia" OR "Pelvic Girdle Pain" OR "Neck Pain" OR "Piriformis Muscle Syndrome" OR "Pudendal Neuralgia" OR "Sciatica" OR "Nociceptive Pain" OR "Intractable Pain" OR "Postoperative Pain" OR "Phantom Limb" OR "Procedural Pain" OR "Referred Pain")) OR ( ("Pelvic Pain" OR "tendinopat*" OR "Enthesopathy" OR "tendon entrapment*" OR "De Quervain" OR "trigger finger" OR "Tenosynovitis" OR "Fibromyalgia" OR "acute pain" OR "Arthralgia" OR "Shoulder Pain" OR "Back Pain" OR "Failed Back Surgery" OR "Low Back Pain" OR "Breakthrough Pain" OR "Facial Pain")) OR ( ("Headache" OR "Metatarsalgia" OR "Mortons Neuroma*" OR "Myalgia")) OR ( ("Pelvic Girdle Pain" OR "Neck Pain" OR "Piriformis Muscle Syndrome" OR "Pudendal Neuralgia" OR "Sciatica")) OR ( ("Nociceptive Pain" OR "Intractable pain" OR "Postoperative Pain" OR "Phantom Limb" OR "Procedural Pain" OR "Phantom Limb" OR "Procedural Pain" OR "Referred Pain" OR "Pelvic Pain" OR "TMJ")) AND (( ("Delivery of Health Care" OR "Attitude of Health Personnel" OR "Emotional Intelligence" OR "Health Personnel" OR "Clinical Reasoning" OR "Judgement" OR "Trust" OR "Professional-Patient Relations" OR "Caregivers" OR "Clinical Decision-Making" OR "healthcare" OR "attitude" OR "empathy" OR "self-motivation" OR "self-awareness" OR "self-regulation" OR "social skill*" OR "nurse*" OR "nursing staff" OR "Doctor*" OR "physical therapist*" OR "physician" OR "occupational therapist" OR "Judgement" OR "trust" OR "Professional Patient Relationship" OR "bias") AND ("Sex Factors" OR "Sexism" OR "Gender Equity" OR "Prejudice" OR "Gender Equity" OR "Gender Identity" OR "Sex Characteristics" OR "Sexuality" OR "Gender Identity" OR "Sex" OR "sexism" OR "gender" OR "Healthcare Disparities") AND ("Pain Management" OR "Pain therap*" OR "Pain treatment*")))

**CINAHL Complete**

( "( "“Delivery of Health Care” OR ”Attitude of Health Personnel” OR ”Emotional Intelligence” OR ”Health Personnel” OR ”Clinical Reasoning” OR ”Judgement” OR ”Trust” OR ”Professional-Patient Relations” OR "Caregivers" OR "Clinical Decision-Making" OR ”healthcare” OR ”attitude” OR ”empathy” OR ”self-motivation” OR “self-awareness” OR “self-regulation” OR “social skill*” OR ”nurse*” OR “nursing staff” OR ”Doctor*” OR “physical therapist*” OR ”physician” OR “occupational therapist” OR ”Judgement” OR ”trust” OR “Professional Patient Relationship” OR ”bias”" ) AND ( ”Sex Factors” OR ”Sexism” OR ”Gender Equity” OR ”Prejudice” OR ”Gender Equity” OR ”Gender Identity” OR "Sex Characteristics" OR "Sexuality" OR "Gender Identity" OR "Sex" OR ”sexism” OR ”gender” OR "Healthcare Disparities" ) AND ( "Pain Management" OR “Pain therap*” OR “Pain treatment*” )" ) AND ( “Chronic Pain” OR “Musculoskeletal pain*” OR "Craniomandibular Disorders" OR "Temporomandibular Joint Disorders" OR "Temporomandibular Joint Dysfunction Syndrome" OR "Medial Tibial Stress Syndrome" OR "Myofascial Pain Syndromes" OR "Temporomandibular Joint Dysfunction Syndrome" OR "Tendinopathy" OR "Elbow Tendinopathy" OR "Tennis Elbow" OR "Enthesopathy" OR "Tendon Entrapment" OR "De Quervain Disease" OR "Trigger Finger Disorder" OR "Tenosynovitis" OR "Fibromyalgia" OR “pain” OR "Acute Pain" OR "Arthralgia" OR "Shoulder Pain" OR "Back Pain" OR "Failed Back Surgery Syndrome" OR "Low Back Pain" OR "Breakthrough Pain" OR "Facial Pain" OR "Headache" OR "Metatarsalgia" OR "Mortons Neuroma" OR "Myalgia" OR "Pelvic Girdle Pain" OR "Neck Pain" OR "Piriformis Muscle Syndrome" OR "Pudendal Neuralgia" OR "Sciatica" OR "Nociceptive Pain" OR "Intractable Pain" OR "Postoperative Pain" OR "Phantom Limb" OR "Procedural Pain" OR "Referred Pain" OR "Pelvic Pain" OR ”tendinopat*” OR ”Enthesopathy” OR “tendon entrapment*” OR “De Quervain” OR “trigger finger” OR ”Tenosynovitis” OR ”Fibromyalgia” OR “acute pain” OR "Arthralgia” OR "Shoulder Pain" OR "Back Pain" OR "Failed Back Surgery” OR “Low Back Pain" OR “Breakthrough Pain" OR "Facial Pain" OR "Headache" OR "Metatarsalgia" OR "Mortons Neuroma*" OR "Myalgia" OR "Pelvic Girdle Pain" OR "Neck Pain" OR "Piriformis Muscle Syndrome" OR "Pudendal Neuralgia" OR "Sciatica" OR "Nociceptive Pain" OR "Intractable pain" OR "Postoperative Pain" OR "Phantom Limb" OR "Procedural Pain" OR "Phantom Limb" OR "Procedural Pain" OR "Referred Pain" OR “Pelvic Pain” OR “TMJ” )(Search as Keyword)

**Academic Search Complete**

( "Pain Management" OR “Pain therap*” OR “Pain treatment*” ) AND ( ”Sex Factors” OR ”Sexism” OR ”Gender Equity” OR ”Prejudice” OR ”Gender Equity” OR ”Gender Identity” OR "Sex Characteristics" OR "Sexuality" OR "Gender Identity" OR "Sex" OR ”sexism” OR ”gender” OR "Healthcare Disparities" ) AND ( “Delivery of Health Care” OR ”Attitude of Health Personnel” OR ”Emotional Intelligence” OR ”Health Personnel” OR ”Clinical Reasoning” OR ”Judgement” OR ”Trust” OR ”Professional-Patient Relations” OR "Caregivers" OR "Clinical Decision-Making" OR ”healthcare” OR ”attitude” OR ”empathy” OR ”self-motivation” OR “self-awareness” OR “self-regulation” OR “social skill*” OR ”nurse*” OR “nursing staff” OR ”Doctor*” OR “physical therapist*” OR ”physician” OR “occupational therapist” OR ”Judgement” OR ”trust” OR “Professional Patient Relationship” OR ”bias” ) AND ( “Chronic Pain” OR “Musculoskeletal pain*” OR "Craniomandibular Disorders" OR "Temporomandibular Joint Disorders" OR "Temporomandibular Joint Dysfunction Syndrome" OR "Medial Tibial Stress Syndrome" OR "Myofascial Pain Syndromes" OR "Temporomandibular Joint Dysfunction Syndrome" OR "Tendinopathy" OR "Elbow Tendinopathy" OR "Tennis Elbow" OR "Enthesopathy" OR "Tendon Entrapment" OR "De Quervain Disease" OR "Trigger Finger Disorder" OR "Tenosynovitis" OR "Fibromyalgia" OR “pain” OR "Acute Pain" OR "Arthralgia" OR "Shoulder Pain" OR "Back Pain" OR "Failed Back Surgery Syndrome" OR "Low Back Pain" OR "Breakthrough Pain" OR "Facial Pain" OR "Headache" OR "Metatarsalgia" OR "Mortons Neuroma" OR "Myalgia" OR "Pelvic Girdle Pain" OR "Neck Pain" OR "Piriformis Muscle Syndrome" OR "Pudendal Neuralgia" OR "Sciatica" OR "Nociceptive Pain" OR "Intractable Pain" OR "Postoperative Pain" OR "Phantom Limb" OR "Procedural Pain" OR "Referred Pain" OR "Pelvic Pain" OR ”tendinopat*” OR ”Enthesopathy” OR “tendon entrapment*” OR “De Quervain” OR “trigger finger” OR ”Tenosynovitis” OR ”Fibromyalgia” OR “acute pain” OR "Arthralgia” OR "Shoulder Pain" OR "Back Pain" OR "Failed Back Surgery” OR “Low Back Pain" OR “Breakthrough Pain" OR "Facial Pain" OR "Headache" OR "Metatarsalgia" OR "Mortons Neuroma*" OR "Myalgia" OR "Pelvic Girdle Pain" OR "Neck Pain" OR "Piriformis Muscle Syndrome" OR "Pudendal Neuralgia" OR "Sciatica" OR "Nociceptive Pain" OR "Intractable pain" OR "Postoperative Pain" OR "Phantom Limb" OR "Procedural Pain" OR "Phantom Limb" OR "Procedural Pain" OR "Referred Pain" OR “Pelvic Pain” OR “TMJ” )

**Rehab Reference Center**

"pain management" AND ("Sex Factors” OR ”Sexism” OR ”Gender Equity” OR ”Prejudice” OR ”Gender Equity” OR ”Gender Identity” OR "Sex Characteristics" OR "Sexuality" OR "Gender Identity" OR "Sex" OR ”sexism” OR ”gender” OR "Healthcare Disparities") AND (“Delivery of Health Care” OR ”Attitude of Health Personnel” OR ”Emotional Intelligence” OR ”Health Personnel” OR ”Clinical Reasoning” OR ”Judgement” OR ”Trust” OR ”Professional-Patient Relations” OR "Caregivers" OR "Clinical Decision-Making" OR ”healthcare” OR ”attitude” OR ”empathy” OR ”self-motivation” OR “self-awareness” OR “self-regulation” OR “social skill*” OR ”nurse*” OR “nursing staff” OR ”Doctor*” OR “physical therapist*” OR ”physician” OR “occupational therapist” OR ”Judgement” OR ”trust” OR “Professional Patient Relationship” OR ”bias” ) AND (“Chronic Pain” OR “Musculoskeletal pain*” OR "Craniomandibular Disorders" OR "Temporomandibular Joint Disorders" OR "Temporomandibular Joint Dysfunction Syndrome" OR "Medial Tibial Stress Syndrome" OR "Myofascial Pain Syndromes" OR "Temporomandibular Joint Dysfunction Syndrome" OR "Tendinopathy" OR "Elbow Tendinopathy" OR "Tennis Elbow" OR "Enthesopathy" OR "Tendon Entrapment" OR "De Quervain Disease" OR "Trigger Finger Disorder" OR "Tenosynovitis" OR "Fibromyalgia" OR “pain” OR "Acute Pain" OR "Arthralgia" OR "Shoulder Pain" OR "Back Pain" OR "Failed Back Surgery Syndrome" OR "Low Back Pain" OR "Breakthrough Pain" OR "Facial Pain" OR "Headache" OR "Metatarsalgia" OR "Mortons Neuroma" OR "Myalgia" OR "Pelvic Girdle Pain" OR "Neck Pain" OR "Piriformis Muscle Syndrome" OR "Pudendal Neuralgia" OR "Sciatica" OR "Nociceptive Pain" OR "Intractable Pain" OR "Postoperative Pain" OR "Phantom Limb" OR "Procedural Pain" OR "Referred Pain" OR "Pelvic Pain" OR ”tendinopat*” OR ”Enthesopathy” OR “tendon entrapment*” OR “De Quervain” OR “trigger finger” OR ”Tenosynovitis” OR ”Fibromyalgia” OR “acute pain” OR "Arthralgia” OR "Shoulder Pain" OR "Back Pain" OR "Failed Back Surgery” OR “Low Back Pain" OR “Breakthrough Pain" OR "Facial Pain" OR "Headache" OR "Metatarsalgia" OR "Mortons Neuroma*" OR "Myalgia" OR "Pelvic Girdle Pain" OR "Neck Pain" OR "Piriformis Muscle Syndrome" OR "Pudendal Neuralgia" OR "Sciatica" OR "Nociceptive Pain" OR "Intractable pain" OR "Postoperative Pain" OR "Phantom Limb" OR "Procedural Pain" OR "Phantom Limb" OR "Procedural Pain" OR "Referred Pain" OR “Pelvic Pain” OR “TMJ”)
