# Supplementary material for: International perspective on healthcare provider gender bias in musculoskeletal pain management: a scoping review
Source: BMJ Open. 2026 Jan 12;16(1):e107766. doi: 10.1136/bmjopen-2025-107766 (PMC12815234; doi:10.1136/bmjopen-2025-107766)
Supplement: Supplementary file 4 [file bmjopen-2025-107766.reviewer_comments.pdf]

PEER REVIEW HISTORY

BMJ Open publishes all reviews undertaken for accepted manuscripts. Reviewers are asked to complete a checklist review form and are provided with free text boxes to elaborate on their assessment. These free text comments are reproduced below.

ARTICLE DETAILS

Title (Provisional)

International Perspective on Healthcare Provider Gender Bias in Musculoskeletal Pain Management: A Scoping Review

Authors

Wilford, Katherine F.; Mena-Iturriaga, Maria Jesus; Vugrin, Margaret; Wainer, Macarena; Seeber, Gesine H.

VERSION 1 - REVIEW

**Reviewer**	**1**
**Name**	**Gholamrezaei, Ali**
**Affiliation**	**The University of Sydney**
**Date**	**19-Sep-2025**
**COI**	**None**

This scoping review aimed to systematically identify and map the available scientific and grey literature on healthcare provider gender bias in the assessment, diagnosis, and management of musculoskeletal pain, as well as to identify gaps that necessitate further research. The review was conducted according to best practice guidelines, and the protocol was prospectively registered and published in a peer-reviewed journal.

Major Comment

The authors conclude that "a true gender bias in the management of patients with MSK pain is apparent." Scoping reviews are designed to map evidence, not to establish certainty of findings. Unlike systematic reviews and meta-analyses, they do not typically include a critical appraisal of evidence quality and therefore cannot make definitive statements about causality or certainty in evidence. I recommend revising the conclusion to more cautiously reflect the evidence.

Minor Comments

The result section in the abstract is currently too brief. I recommend shortening the background and expanding the results with key findings.

The authors list as a strength that the team comprises “an international group of early-, mid-, and late-career researchers.” While having multilingual expertise is clearly a strength, it is unclear how career stage diversity directly enhances review quality in an objective sense. Review teams should include people with the right skills and subject expertise (e.g., topic experts, methodologists, statisticians if relevant, librarians). Instead of this statement, Authors can highlight other clear strengths of their review, for example that the protocol was peer-reviewed and published, etc.

Instead of stating “Covidence was used during the screening and data extraction process to ensure blinding reviewer consistency,” I suggest rephrasing to emphasize adherence to best-practice guidelines in conducting and reporting scoping reviews.

**Reviewer**	**2**
**Name**	**Hlongwa, Mbuzeleni**
**Affiliation**	**University of KwaZulu-Natal College of Health Sciences, Nursing and Public Health**
**Date**	**09-Oct-2025**
**COI**	**None**

Thank you for the opportunity to review this manuscript. I appreciated the opportunity to read and evaluate this work. However, I found that the manuscript contains several fundamental methodological and reporting flaws, including numerous numerical inconsistencies in the PRISMA flow diagram and an insufficiently detailed Methods section, which collectively undermine confidence in the study’s findings. These issues raise serious concerns about the replicability, transparency, and overall integrity of the review process.

Major concerns

• The manuscript repeatedly refers to “PRISMA-SR.” The correct guideline for scoping reviews is PRISMA-ScR.

• The phrase “from inception to August 2022 and updated in May 2025” is ambiguous. If the original search was August 2022, the update in May 2025 requires explicit justification (why the long gap?), explicit dates when each database was re-searched, and confirmation that the same search strategies were used.

• The only inclusion criterion reported appears to be language: “published in English, Spanish, or German.” That is insufficient. A scoping review must state clear, detailed criteria — at minimum: population (or concept), types of evidence (peer-reviewed studies, grey literature, conference abstracts), settings, study designs included/excluded, date limits (if any), and other eligibility filters. The authors should present these and justify language restrictions (or acknowledge resulting bias).

• PRISMA-ScR expects detailed methods to allow reproducibility. Specific deficiencies:

o The authors did not list or define the variables they extracted (e.g., year, country, study design, population characteristics, sample size, interventions/exposures, outcome measures, key findings, funding/conflicts). PRISMA-ScR requires these to be listed and defined.

o No description of the charting form, whether it was piloted, how many reviewers extracted data, or how discrepancies were resolved, although this was discussed for article screenings.

o The manuscript fails to describe how charted data were handled and summarized (narrative synthesis? thematic analysis? evidence mapping? frequency counts?). Without this information I could judge how conclusions were reached.

o While scoping reviews may omit risk-of-bias assessment, the authors must at least state their decision not to conduct this and justify it. I fully understand that this step is not compulsory, however, it is also recommended.

• The PRISMA flow diagram contains multiple numerical inconsistencies.

o Authors state: 4965 articles retrieved from different sources and 350 from other sources, minus 1329 duplicates → by arithmetic:

▯ 4965 + 350 = 5315

▯ 5315 − 1329 = 3986

▯ The authors report 3941 screened. Discrepancy = 3941 vs 3986 (difference 45).

▯ Authors report: screened 3941; after screening removed 3605 → 3941 − 3605 = 336, not the 337 reported by the authors. (Off by 1.)

▯ “References from other sources: n = 350” — but the breakdown shown (10 + 144 + 151) sums to 305, not 350. (Again difference = 45.)

▯ Provide exact hit counts for every searched source (database name + number of hits for that search).

▯ Provide a corrected PRISMA flow diagram with exact arithmetic and a table of per-source counts as an appendix.

▯ Replace “unspecified” under Identification with explicit names of all databases and grey literature sources searched — “unspecified” is not acceptable.

o Appendix A: While the manuscript may include search strings, it must also report the number of articles retrieved by each strategy/database on the date searched. This permits verification and allows readers to reproduce/assess the search comprehensively.

Minor issues

• The authors must justify language restrictions (English/Spanish/German) and discuss the risk of language bias in Limitations.

• Provide or register a study protocol (e.g., OSF).

• Improve writing and correct typos (e.g., consistent spelling of PRISMA-ScR, editorial proofreading).

VERSION 1 - AUTHOR RESPONSE

REVIEWER #1 COMMENTS

This scoping review aimed to systematically identify and map the available scientific and grey literature on healthcare provider gender bias in the assessment, diagnosis, and management of musculoskeletal pain, as well as to identify gaps that necessitate further research. The review was conducted according to best practice guidelines, and the protocol was prospectively registered and published in a peer-reviewed journal.

> **Author Response:** Thank you for reviewing our manuscript. We addressed all your comments below.

Comment 1:

The authors conclude that "a true gender bias in the management of patients with MSK pain is apparent." Scoping reviews are designed to map evidence, not to establish certainty of findings. Unlike systematic reviews and meta-analyses, they do not typically include a critical appraisal of evidence quality and therefore cannot make definitive statements about causality or certainty in evidence. I recommend revising the conclusion to more cautiously reflect the evidence.

> **Author Response**: Thank you for this feedback. We have modified this part of the Conclusion Section to be more consistent with the global purpose/design of scoping reviews. The modified sentence has been copied below for your convenience.
>
> > *While there appears to be inconsistent use of the terms sex and gender, the literature informing this review suggests an existence of gender bias in the management of patients with MSK pain. [Lines 54-55 and 396-397]*

Comment 2:

The result section in the abstract is currently too brief. I recommend shortening the background and expanding the results with key findings.

> **Author Response:** Thank you for this suggestion. The abstract has been edited accordingly to allow for more information in the Results section.

Comment 3:

The authors list as a strength that the team comprises "an international group of early-, mid-, and late-career researchers." While having multilingual expertise is clearly a strength, it is unclear how career stage diversity directly enhances review quality in an objective sense. Review teams should include people with the right skills and subject expertise (e.g., topic experts, methodologists, statisticians if relevant, librarians). Instead of this statement, Authors can highlight other clear strengths of their review, for example that the protocol was peer-reviewed and published, etc.

> **Author Response:** Thank you for this feedback. We have edited the Strengths and Limitations section and copied it below for review.
>
> - *The research team comprises an international group of researchers as well as a*

scientific librarian with expertise in developing search strategies

- *The protocol for this scoping review has been previously peer reviewed and published*

Comment 4:

Instead of stating "Covidence was used during the screening and data extraction process to ensure blinding reviewer consistency," I suggest rephrasing to emphasize adherence to best-practice guidelines in conducting and reporting scoping reviews.

Author Response: Thank you for this suggestion. We have modified this sentence and copied below for your review.

The web-based collaboration software platform (Covidence) was used during the screening and data extraction processes for optimal adherence to best practice guidelines in the conducting and reporting of scoping reviews

REVIEWER #2 COMMENTS:

Comment 1:

Thank you for the opportunity to review this manuscript. I appreciated the opportunity to read and evaluate this work. However, I found that the manuscript contains several fundamental methodological and reporting flaws, including numerous numerical inconsistencies in the PRISMA flow diagram and an insufficiently detailed Methods section, which collectively undermine confidence in the study's findings. These issues raise serious concerns about the replicability, transparency, and overall integrity of the review process.

Author Response: Thank you for your time and expertise in reviewing this manuscript. Your feedback will serve to improve the quality of the paper and assist with the dissemination of this important topic.

Comment 2:

The manuscript repeatedly refers to "PRISMA-SR." The correct guideline for scoping reviews is PRISMA-ScR.

Author Response: Thank you for bringing this to our attention. We have corrected this abbreviation in the abstract [Line 32] as well as in Line 148.

Comment 3:

The phrase "from inception to August 2022 and updated in May 2025" is ambiguous. If the original search was August 2022, the update in May 2025 requires explicit justification (why the long gap?), explicit dates when each database was re-searched, and confirmation that the same search strategies were used.

Author Response: Thank you for this feedback. We have added justification for the time between searches [Lines 157-159]. Additionally, we have added the exact dates when each search was run [Lines 152 and 159]

Comment 4:

The only inclusion criterion reported appears to be language: "published in English, Spanish, or German." That is insufficient. A scoping review must state clear, detailed criteria — at minimum: population (or concept), types of evidence (peer-reviewed studies, grey literature, conference

abstracts), settings, study designs included/excluded, date limits (if any), and other eligibility filters. The authors should present these and justify language restrictions (or acknowledge resulting bias).

Author Response: Thank you for bringing this to our attention. We have added language to further expand on our inclusion criteria. This has been copied below for review.

> *References from scientific literature and gray literature were included if they involved human participants with musculoskeletal pain and examined HCP-GB as the dependent variable. All methodologies were included; however, reviews/meta-analyses were excluded from data extraction to avoid extracting duplicate data. [Lines 166-170]*

Comment 5:

PRISMA-ScR expects detailed methods to allow reproducibility. Specific deficiencies:

o The authors did not list or define the variables they extracted (e.g., year, country, study design, population characteristics, sample size, interventions/exposures, outcome measures, key findings, funding/conflicts). PRISMA-ScR requires these to be listed and defined.

o No description of the charting form, whether it was piloted, how many reviewers extracted data, or how discrepancies were resolved, although this was discussed for article screenings.

o The manuscript fails to describe how charted data were handled and summarized (narrative synthesis? thematic analysis? evidence mapping? frequency counts?). Without this information, I could judge how conclusions were reached.
o While scoping reviews may omit risk-of-bias assessment, the authors must at least state their decision not to conduct this and justify it. I fully understand that this step is not compulsory, however, it is also recommended.

Author Response: Thank you for this feedback. Table 2 is a detailed overview of the variables extracted as well as the reporting/charting methodology for each variable. If additional information is requested, please provide further clarification.

Regarding the pilot process and the number of reviewers involved, this process is described in the Methods Section and copied below for review.

> *The review management software Covidence (Veritas Health Innovation Ltd, Melbourne, Australia) was used for article screening, selection, and data extraction. Before starting the final review, two pilot screenings were incorporated, where the first aimed to define and revise the search criteria and the second at refining article selection and data extraction processes. (33,35,40) A three-reviewer model was employed throughout the entire article selection process, where two reviewers ([redacted]) independently and blinded to the other reviewer's vote, screened each item. In case of conflicts between both reviewers' votes, a third reviewer ([redacted]) resolved these. [Lines 176-183]*

Regarding the risk-of-bias assessment, according to the PRISMA-ScR guidelines, if this bias assessment is performed, a rationale should be provided to support the decision. In the current scoping review, however, no risk-of-bias assessment was performed; therefore, no mention/justification of the assessment is required. While we understand the reviewer's

comment on this assessment being recommended, it is currently not best-practice for the completion of scoping reviews.

Comment 6:

The PRISMA flow diagram contains multiple numerical inconsistencies. Authors state: 4965 articles retrieved from different sources and 350 from other sources, minus 1329 duplicates → by arithmetic: 4965 + 350 = 5315 and 5315 − 1329 = 3986

The authors report 3941 screened. Discrepancy = 3941 vs 3986 (difference 45).

Authors report: screened 3941; after screening removed 3605 → 3941 − 3605 = 336, not the 337 reported by the authors. (Off by 1.)

“References from other sources: n = 350” — but the breakdown shown (10 + 144 + 151) sums to 305, not 350. (Again difference = 45.)

Provide exact hit counts for every searched source (database name + number of hits for that search).

Provide a corrected PRISMA flow diagram with exact arithmetic and a table of per-source counts as an appendix.

Replace “unspecified” under Identification with explicit names of all databases and grey literature sources searched — “unspecified” is not acceptable.

Author Response: Thank you for bringing this to our attention. We have verified our original data and corrected the numbers in the PRIMSA flow diagram, so the math is correct. Additionally, we have removed the “unspecified” category and correctly allocated those references to the databases they were originally retrieved from.
Since the numbers in the PRISMA flow diagram are now accurate and reflect each database and the number of references retrieved, we feel a table with these same data would be redundant.

Comment 7:

Appendix A: While the manuscript may include search strings, it must also report the number of articles retrieved by each strategy/database on the date searched. This permits verification and allows readers to reproduce/assess the search comprehensively

Author Response: Thank you for this comment. In our corrected flowchart (Figure 1) we have reported the number of articles retrieved from each database.

Comment 8:

The authors must justify language restrictions (English/Spanish/German) and discuss the risk of language bias in Limitations.

Author Response: Thank you for this feedback. We have added language to the Methods section as further justification for why the language restrictions were used.

Due to the skills of the author team, resources were deemed eligible if they were published in English, Spanish, or German. [Lines 165-166]

The possible risk of these restrictions has been previously addressed in the Limitations section. If additional modifications are requested, please advise further.

> *First, only references in English, Spanish, or German were included. This may have inadvertently excluded a wider variety of studies that could have improved the diversity of findings. [Lines 381-383]*

Comment 9:
Provide or register a study protocol (e.g., OSF).

Author Response: Thank you for this feedback. Instead of registering this project, the full protocol for this scoping review has been previously published and referenced in the Methods section [Line 148-149]. This reference has been coped below for your review.

> *Wilford KF, Mena-Iturriaga MJ, Vugrin M, Wainer M, Sizer PS, Seeber GH. International perspective on healthcare provider gender bias in musculoskeletal pain management: a scoping review protocol. BMJ Open. 2022;12(6):e059233.*

Comment 10:
Improve writing and correct typos (e.g., consistent spelling of PRISMA-ScR, editorial proofreading).

Author Response: Thank you for this feedback. As mentioned previously, we have corrected the abbreviation PRISMA-ScR in the abstract and on Line 148. Additionally, the entire manuscript has undergone extensive editorial proofreading prior to submission.
